# Anti-cancer effect of bee venom on colon cancer cell growth by activation of death receptors and inhibition of nuclear factor kappa B

**DOI:** 10.18632/oncotarget.6295

**Published:** 2015-10-30

**Authors:** Jie Zheng, Hye Lim Lee, Young Wan Ham, Ho Sueb Song, Min Jong Song, Jin Tae Hong

**Affiliations:** ^1^ College of Pharmacy and Medical Research Center, Chungbuk National University, Heungduk-gu, Cheongju, Chungbuk 361-763, South Korea; ^2^ Department of Chemistry, Utah Valley University, Orem, UT 84508, USA; ^3^ College of Oriental Medicine, Kyungwon University, Sujeong-gu, Seongnam, Gyeonggii 461-701, South Korea; ^4^ Department of Obstetrics and Gynecology, Daejeon St. Mary's Hospital, College of Medicine, The Catholic University of Korea, Jung-gu, Daejeon 301-723, South Korea

**Keywords:** bee venom, NF-κB, death receptor, p50, colon cancer

## Abstract

Bee venom (BV) has been used as a traditional medicine to treat arthritis, rheumatism, back pain, cancerous tumors, and skin diseases. However, the effects of BV on the colon cancer and their action mechanisms have not been reported yet. We used cell viability assay and soft agar colony formation assay for testing cell viability, electro mobility shift assay for detecting DNA binding activity of nuclear factor kappa B (NF-κB) and Western blotting assay for detection of apoptosis regulatory proteins. We found that BV inhibited growth of colon cancer cells through induction of apoptosis. We also found that the expression of death receptor (DR) 4, DR5, p53, p21, Bax, cleaved caspase-3, cleaved caspase-8, and cleaved caspase-9 was increased by BV treatment in a dose dependent manner (0–5 μg/ml). Consistent with cancer cell growth inhibition, the DNA binding activity of nuclear factor kappa B (NF-κB) was also inhibited by BV treatment. Besides, we found that BV blocked NF-κB activation by directly binding to NF-κB p50 subunit. Moreover, combination treatment with BV and p50 siRNA or NF-κB inhibitor augmented BV-induced cell growth inhibition. However, p50 mutant plasmid (C62S) transfection partially abolished BV-induced cell growth inhibiton. In addition, BV significantly suppressed tumor growth *in vivo*. Therefore, these results suggested that BV could inhibit colon cancer cell growth, and these anti-proliferative effects may be related to the induction of apoptosis by activation of DR4 and DR5 and inhibition of NF-κB.

## INTRODUCTION

Colon cancer is one of the major causes of cancer-related deaths in the world [1]. Colon cancer has an estimated incidence of over 1,000,000 new cases annually worldwide. Almost one of three patients with colon cancer dies from the disease. Colon cancer also more often affects people of well-developed countries in comparison to less developed countries [2]. Several epidemiological and laboratory studies have demonstrated the association of colon cancer with environmental factors such as western style dietary habits, low fiber intake, tobacco-smoking, high fat diet, low calcium/micronutrient intake and lack of physical activities [3]. However, there are also negative studies on fat, fiber and calcium in colon cancer development [4]. In addition, colon cancer is a heterogeneous disease. There is evidence that lifestyle factors like diet can modulate the course of this disease [5].

Constitutive activation of Nuclear Factor-κB (NF-κB) has been described in a great number of tumors including colon tumors. Human colon cancer cell lines and tumor samples as well as the nuclei of stromal macrophages in sporadic adenomatous polyps showed increased NF-κB activity [6, 7]. The inhibition of NF-κB has shown remarkable anti-tumor activity in preclinical and clinical studies [8]. NF-κB signaling has a pivotal role in cancer development and progression. NF-κB provides a mechanistic link between inflammation and cancer, and is a major factor controlling the ability of both pre-neoplastic and malignant cells to resist apoptosis-based tumor-surveillance mechanisms [9]. NF-κB might also regulate tumor angiogenesis and invasiveness, and the signaling pathways that adjust its activation provide attractive targets for new chemo-preventive and chemotherapeutic approaches [10]. A number of reports have shown that NF-κB is inhibited by apoptosis-inducing agents in human colon cancer cells [11, 12]. NF-κB acts as a cell survival factor through its regulatory role in the expression of an array of apoptotic (caspase-3 and Bax), antiapoptotic (Bcl-2 and IAP family), and cell proliferation genes (cyclooxygenase-2 and cyclins) [13]. Therefore, inhibition of NF-κB by chemotherapeutics is intended as a new strategy to eliminate cancerous cells through induction of apoptosis.

Tumor necrosis factor-related apoptosis-inducing ligand (TRAIL) induces apoptosis in cancer cells without toxicity to normal cells. It is well known that TRAIL binds to DRs, TRAIL-R1 (DR4) and TRAIL-R2 (DR5) expressed on cancer cell surface and activates apoptotic pathways. When DRs bind to their ligands, the death domains recruit the intracellular adaptor protein (Fas-associated death domain protein) which results in the activation of capase-8 that leads to the activation of downstream caspases, including capase-3, caspase-9 and Bax [14]. It is documented that the resistance of cancer cells is related to the activation of NF-κB and down-regulation of DRs; thus, down-regulation of NF-κB and up-regulation of DRs are implicated in the development of new anti-cancer treatment for chemo-resistant cancer cells. It has been demonstrated that up-regulation of DRs and inactivation of NF-κB are reciprocally associated in chemoresistance. Many chemotherapeutic agents or natural compounds such as garicinol, zerumbone, quercetin, snake venom toxin inhibit cancer cell growth via overexpression of death receptor associated with down-regulation of NF-κB [15–18]. In agreement with this notion, it is possible that BV could overcome resistance to TRAIL through inactivation of NF-κB and activation of DRs.

BV contains a variety of different peptides, including melittin (a major component of BV), apamin, adolapin, and mast cell degranulating peptide [19, 20]. BV has anti-cancer activity [21]. Melittin, a major polypeptide of BV, is thought to function as a lytic agent that has been used traditionally against chronic inflammation and cancer [22] and also used for the therapeutic agent of arthritis, rheumatism, atherosclerosis, and cancer in traditional medicine [23, 24]. Melittin inhibited the DNA-binding and transcriptional activity of NF-κB, an important transcriptional factor regulating inflammatory gene expression, through the suppression of IκB phosphorylation and the decrease in the translocation of the p50 and p65 subunits of NF-κB [25]. However, the anti-cancer effect of BV on colon cancer cells has not been studied yet. In this study, we investigated anti-cancer effects and possible mechanisms of BV on colon cancer cells. In particular, we determined the capacity of the BV induced apoptotic effect through activation of DRs and suppression of NF-κB.

## RESULTS

### Effect of BV on the growth of colon cancer cells

**Figure 1:**
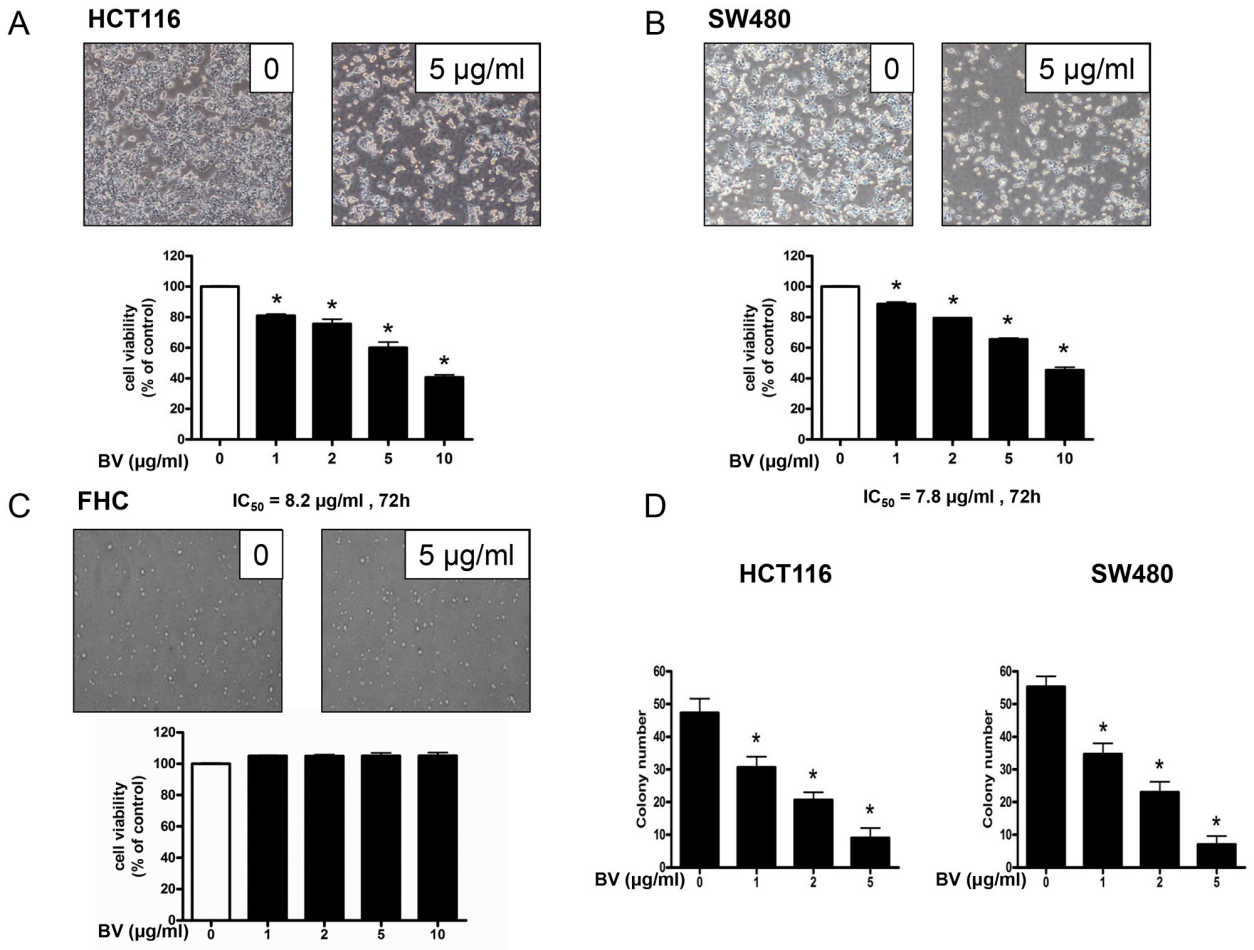
Effect of bee venom (BV) on the growth of colon cancer cells **A, B & C.** Effect of BV on the growth of HCT116 and SW480 colon cancer cells and FHC colon epithelial normal cells. Cell viability was determined by MTT assay. **D.** Effect of BV on the colony formation in HCT116 and SW480 cells. Data was expressed as the mean ± S.D. of three experiments. *p < 0.05 indicates statistically significant differences from control group.

**Figure 2:**
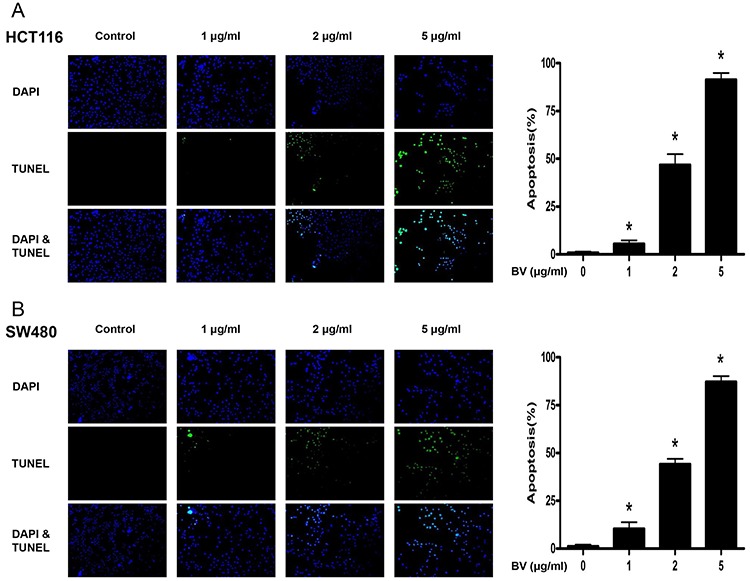
Effect of BV on apoptotic cell death **A.** Apoptotic cell death of HCT116. **B.** Apoptotic cell death of SW480. Colon cancer cells were treated with BV (0–5 μg/ml) for 24 h, and then labeled with DAPI and TUNEL solution. Total number of cells in a given area was determined by using DAPI nuclear staining (fluorescent microscope). A green color in the fixed cells marks TUNEL-labeled cells. Apoptotic index was determined as the DAPI-stained TUNEL-positive cell number / total DAPI-stained cell number × 100%. Data was expressed as the mean ± S.D. of three experiments. *p < 0.05 indicates statistically significant differences from control cells.

To evaluate the effect of BV on the growth of colon cancer cells, we analyzed cell viability using the MTT assay. BV (0–10 μg/ml) inhibited the growth of human colon cancer cells, and the IC_50_ values of HCT116 (Figure 1A) and SW480 (Figure 1B) were 8.2 and 7.8 μg/ml, respectively. We evaluated changes in the morphology of human colon cancer cells using phasecontrast microscopy. BV-treated SW480 cells for 48 h showed rounded morphology and tended to detach from substratum. However, BV-treated HCT116 cells for 48 h presented with membrane blebbing, cell shrinkage, cytoplasmic increasing density and irregularity in shape. (0–10 μg/ml) To evaluate the effect of BV on the colon epithelial normal cells, we treated FHC with BV (0–10 μg/ml) for 24 h, and BV showed no toxicity at all (Figure 1C). To further demonstrate the inhibitory effect of BV on cancer cell growth, we performed the soft agar colony formation assay. The number of colonies was concentration-dependently decreased by BV (Figure 1D), and the concentration of BV for inhibition of colony formation was similar to the cell growth inhibition. To determine whether the inhibition of cell growth by BV treatment was due to induction of apoptosis, we evaluated the changes in colon cancer cells by using DAPI staining followed by TUNEL assays, and then the double labeled cells were analyzed by fluorescence microscope. The cells were treated with BV (0–5 μg/ml) for 24 h. DAPI-stained TUNEL-positive cells were concentration-dependently increased and the highest concentration of BV (5 μg/ml) caused most of cells TUNEL-positive, and apoptosis rates were 91.35% in HCT116 cells (Figure 2A) and 87.23% in SW480 cells (Figure 2B). These results demonstrated that BV treatment strongly induced apoptosis in colon cancer cells.

### Effect of BV on the expression of apoptosis regulatory proteins

**Figure 3:**
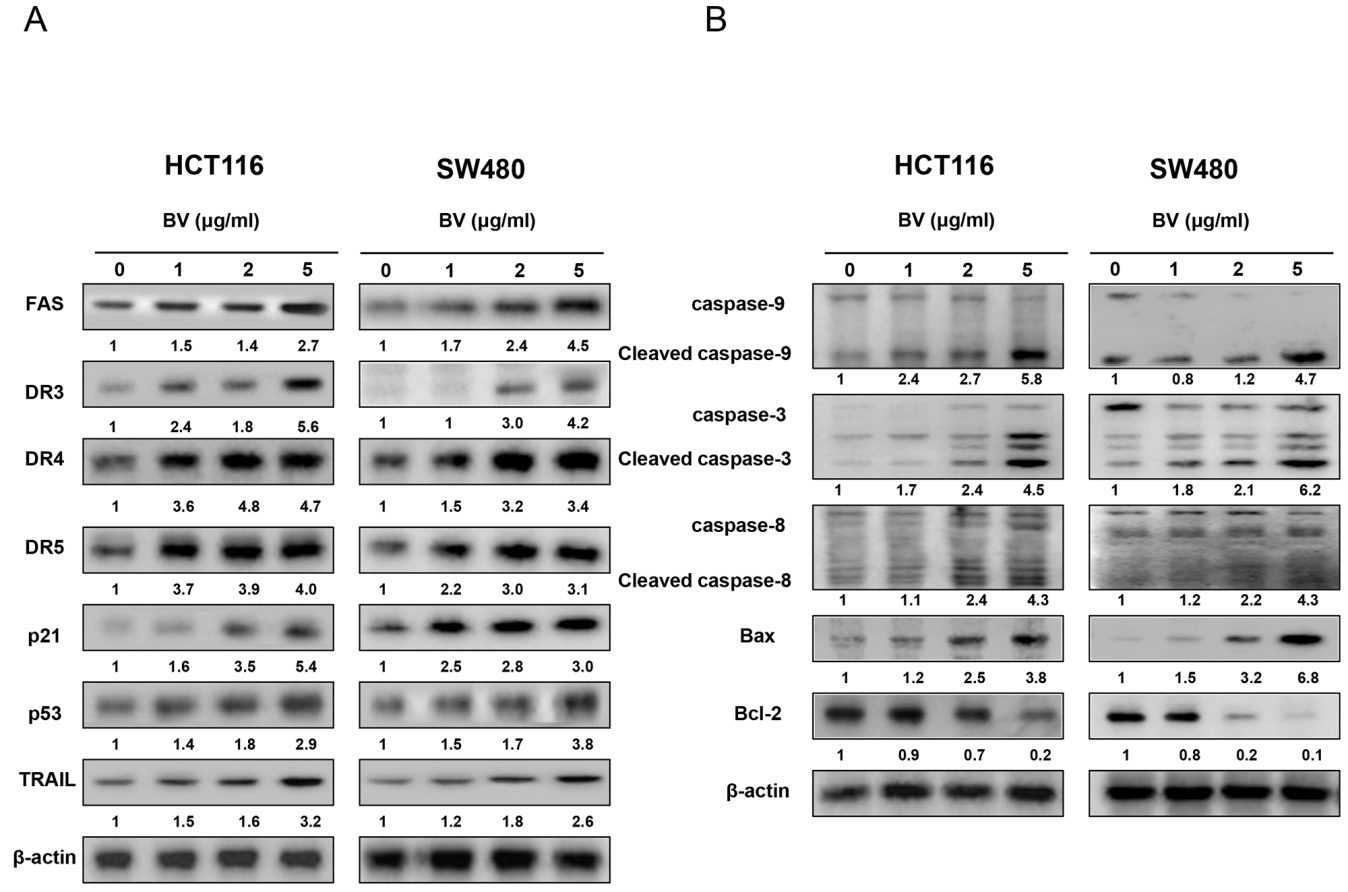
Effect of BV on the expression of apoptosis regulatory proteins **A.** Expression of apoptosis regulatory proteins related intrinsic pathway was determined by Western blotting analysis with antibodies against capase-3, caspase-8, capase-9, Bax, Bcl-2 and β-actin (internal control). **B.** Extrinsic pathway was determined by Western blotting with antibodies against Fas, DR3, DR4, DR5, TRAIL, p21, p53 and β-actin (internal control). Values under Western band indicate the density of band. Each band is representative for three experiments.

To figure out the relationship between the induction of apoptosis and the expression of apoptosis regulatory protein by BV, the expression of apoptosis related intrinsic pathway (Figure 3A) and extrinsic pathway (Figure 3B) proteins was investigated. With the treatment of BV (0–5 μg/ml) in HCT116 and in SW480 colon cancer cells, we found that the expression of pro-apoptotic proteins such as Bax, cleaved caspase-3, cleaved caspase-8 and cleaved caspase-9 as well as the expression of DRs like DR3, DR4, DR5 and Fas was increased in a concentration dependent manner. However, the expression of anti-apoptotic protein Bcl-2 was decreased.

### Effect of BV on NF-κB activation

**Figure 4:**
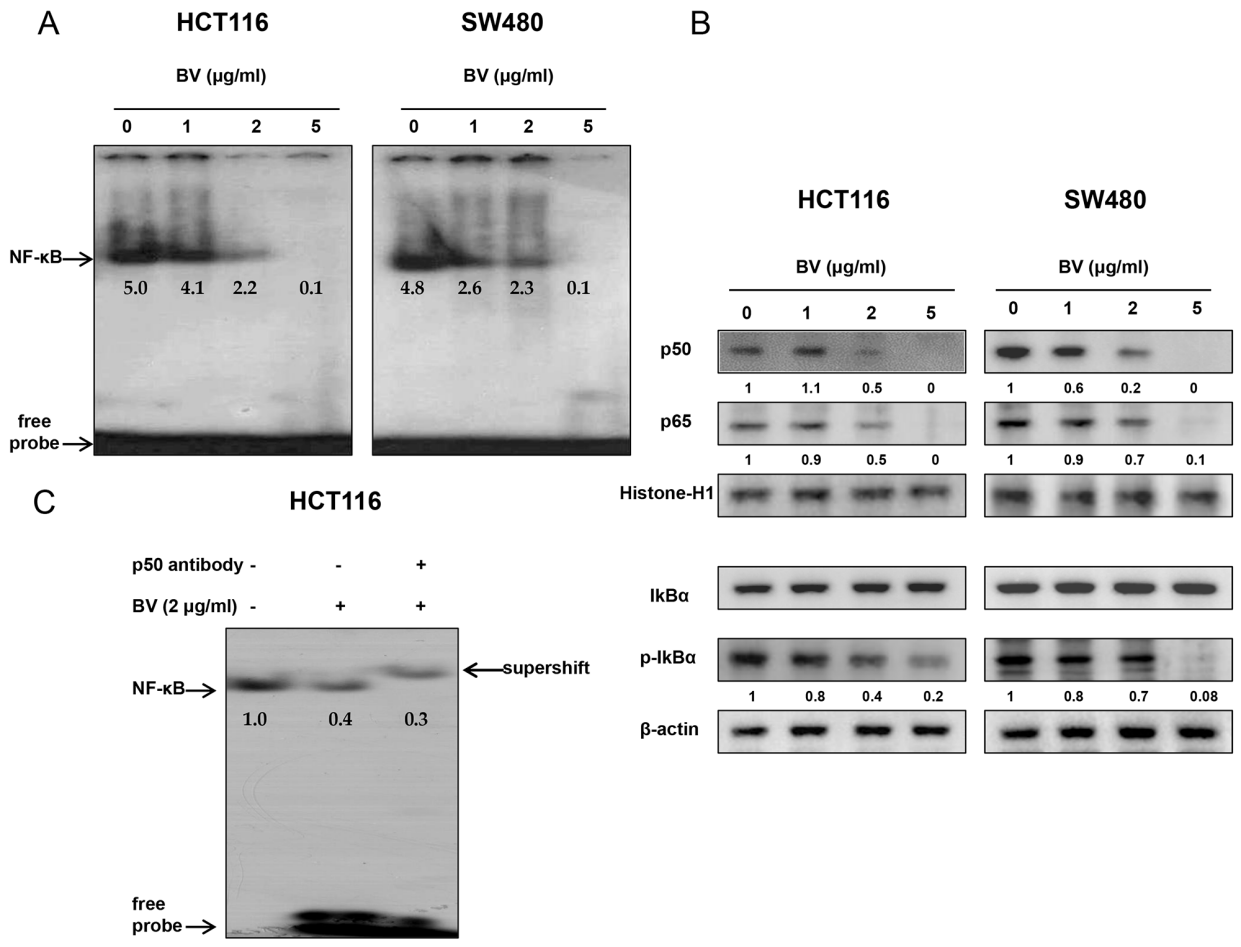
Effect of BV on NF-κB activation in colon cancer cells **A.** Colon cancer cells were treated with BV (0–5 μg/ml) for 2 h, and then were lysed. Nuclear extract was incubated in binding reactions of ^32^p-end-labeled oligonucleotide containing the IκB sequence. The present EMSA results are representative for three experiments. **B.** Cytosolic proteins were used to determine expression of IκB, p-IκB and β-actin (internal control) and nuclear proteins were used to determine expression of p50, p65 and Histone H1 (internal control) in colon cancer cells. Values under Western band indicate the density of band. Each band is representative for three experiments. **C.** Supershift assay was performed on HCT116 cells, and a small volume of p50 antibody (1 μl) was added to the binding mix, and incubated at 37°C for 30 min before loading. The present results are representative for three experiments.

NF-κB plays a significant role in colon cancer cell growth. To investigate whether BV inactivates NF-κB, we performed EMSA for detecting DNA binding activity of NF-κB. We found that BV-untreated colon cancer cells showed highly constituted activation of NF-κB in both colon cancer cells. However, BV treatment concentration dependently inhibited DNA binding activity of NF-κB (Figure 4A). Agreed with the inhibition of NF-κB, cytosolic phosphorylation of IκB as well as the nucleus translocation of p50 and p65 was inhibited by BV treatment in both colon cancer cells (Figure 4B). The band of NF-κB was supershifted by p50 specific antibody in HCT116 colon cancer cells (Figure 4C).

### Reversed effect of DR4 siRNA, DR5 siRNA and TRAIL siRNA on BV-induced cell growth inhibition

**Figure 5:**
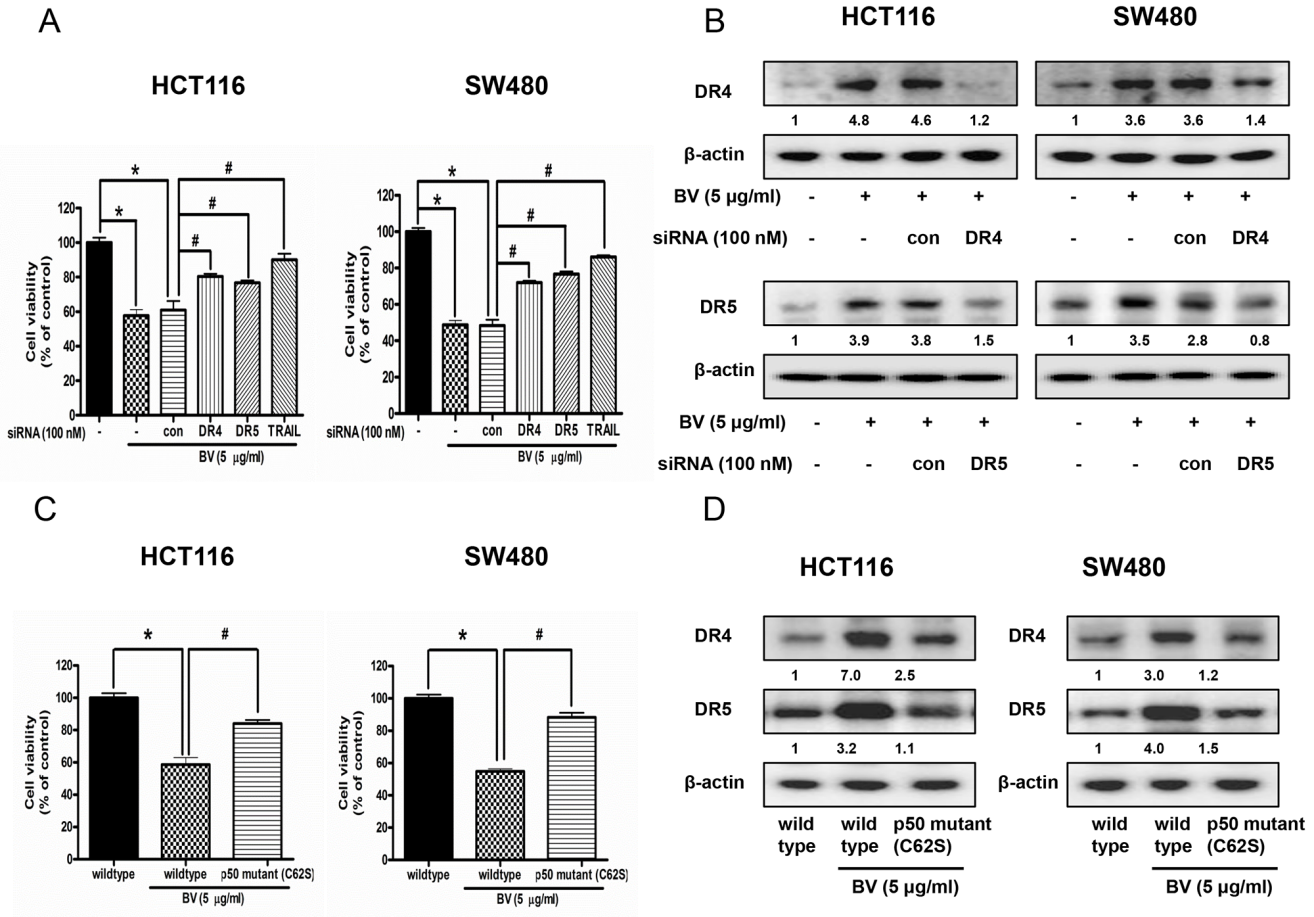
Effect of DR4 siRNA, DR5 siRNA and TRAIL siRNA transfection or p50 mutant plasmid (C62S) transfection on BV-treated colon cancer cell growth and expression of DR4 and DR5 **A.** Colon cancer cells were treated with non-targeting control siRNA and DR4, DR5, TRAIL siRNA (100 nM) for 24 h, and then were treated with BV (5 μg/ml) at 37°C for another 24 h. Cell viability was determined by MTT assay. Data was expressed as the mean ± S.D. of three experiments. *p < 0.05 indicates statistically significant differences from control cells. #*p* < 0.05 indicates significantly different from BV treated cells. **B.** Effect of DR4 siRNA transfection on the expression of DR4 and effect of DR5 siRNA transfection on the expression of DR5. Values under Western band indicate the density of band. Each band is representative for three experiments. **C.** Effect of p50 mutant plasmid (C62S) transfection on BV-treated colon cancer cell growth. Cell viability was determined by MTT assay. Data was expressed as the mean ± S.D. of three experiments. *p < 0.05 indicates statistically significant differences from control cells. #*p* < 0.05 indicates significantly different from BV treated cells. **D.** Effect of p50 mutant plasmid (C62S) transfection on the expression of DR4 and DR5. Cells were transfected by p50 mutant plasmid (C62S) for 24 h, and treated by BV (5 μg/ml) for another 24 h. Whole cell extracts were analyzed by Western blotting using DR4, DR5 and β-actin (internal control) antibodies. Values under Western band indicate the density of band. Each band is representative for three experiments.

To determine the effect of DR4, DR5 and their ligand TRAIL on the BV-induced apoptotic cell death, we performed siRNA transfection and MTT assay. Cells were transfected with DR4 siRNA, DR5 siRNA and TRAIL siRNA by a transfection agent for 24 h, and then were treated with BV (5 μg/ml) for another 24 h. Cell viability was determined by MTT assay. As a result, combination treatment with BV and DR4 or DR5 or TRAIL siRNA transfection partially abolished BV-induced cell growth inhibiton (Figure 5A). Besides, the expression of DR4 was significantly decreased by DR4 siRNA transfection and the expression of DR5 was significantly decreased by DR5 siRNA transfection (Figure 5B).

### Reversed effect of p50 mutant plasmid (C62S) on BV-induced cell growth inhibition and expression of DR4 and DR5

Colon cancer cells were transfected with p50 mutant plasmid (C62S) for 24 h, and then were treated with BV (5 μg/ml) for another 24 h. Cell viability was determined by MTT assay and expression of DR4 and DR5 was detected by Western blotting. As a result, p50 mutant plasmid (C62S) transfection partially abolished BV-induced cell growth inhibiton (Figure 5C), and BV-induced expression of DR4 and DR5 was partially prevented (Figure 5D).

### Synergistic effect of BV and NF-κB inhibitor or p50 siRNA on the growth of human colon cancer cells and expression of DR4 and DR5

**Figure 6:**
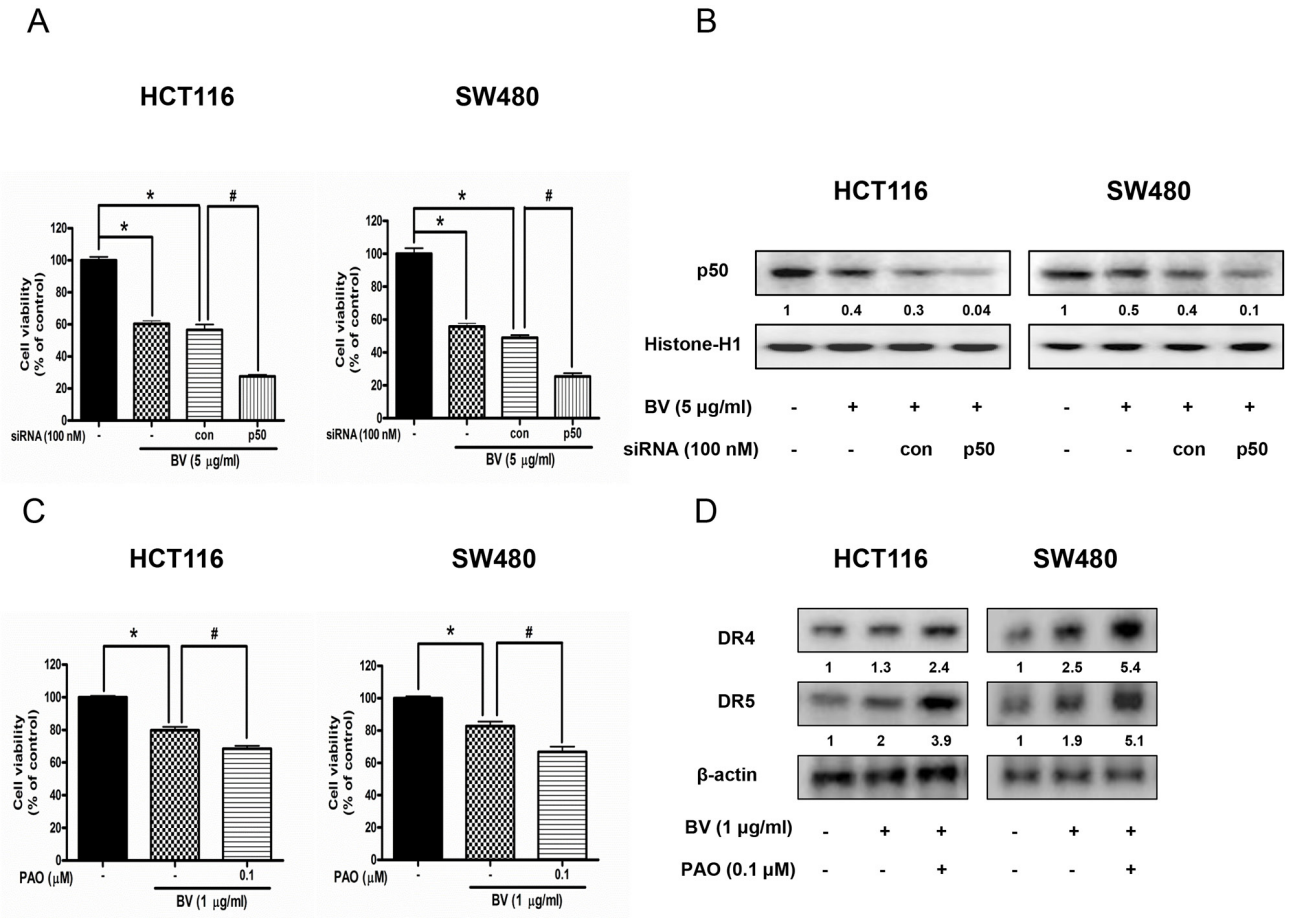
Effect of p50 siRNA transfection or NF-κB inhibitor (PAO) on BV-treated colon cancer cell growth and expression of DR4 and DR5 **A.** Effect of p50 knockdown with p50 siRNA transfection on the inhibitory effect of BV in colon cancer cell growth. Cell viability was determined by MTT assay. *p < 0.05 indicates statistically significant differences from control cells. #*p* < 0.05 indicates significantly different from BV treated cells. **B.** Effect of p50 siRNA transfection on the expression of p50 translocation to nucleus. Values under Western band indicate the density of band. Each band is representative for three experiments. **C.** Effect of NF-κB inhibitor (PAO) on BV-treated colon cancer cell growth. Cells were pretreated with NF-κB inhibitor, PAO (0.1 μM) for 1 h and then were treated with BV for 24 h. Cell viability was determined by MTT assay. *p < 0.05 indicates statistically significant differences from control cells. #*p* < 0.05 indicates significantly different from BV treated cells. **D.** Effect of NF-κB inhibitor (PAO) on the expression of DR4 and DR5. Cells were pretreated with PAO (0.1 μM) for 1 h and then were treated with BV for 24 h, and whole cell extracts were analyzed by Western blotting using DR4, DR5 and β-actin (internal control). Values under Western band indicate the density of band. Each band is representative for three experiments.

To further investigate whether NF-κB plays a critical role in BV-induced up-regulation of DR4 and DR5 as well as colon cancer cell growth inhibition, we pretreated the colon cancer cells with phenylarsine oxide (PAO), an NF-κB inhibitor (0.1 μM) for 1 h, and then these cells were treated with BV (1 μg/ml) for 24 h to assess cell growth, and DR4 and DR5 expression. We also knocked down p50 with p50 siRNA by a transfection agent for 24 h, and then treated the transfected cells with BV (5 μg/ml). Cell viability was determined by MTT assay. As a result, combination treatment with BV and p50 siRNA transfection augmented BV-induced cell growth inhibition (Figure 6A), and the expression of p50 translocation to nucleus was significantly decreased (Figure 6B). Besides, combination treatment with BV and NF-κB inhibitor (PAO) magnified BV-induced cell growth inhibtion (Figure 6C) and enhanced the BV-induced up-regulation of DR4 and DR5 (Figure 6D), suggesting that NF-κB pathway may be involved in BV-induced cell growth inhibition and up-regulation of DR4 and DR5.

### Reversed effect of p53 siRNA or p53 inhibitor on the growth of human colon cancer cells and expression of DR4 and DR5

**Figure 7:**
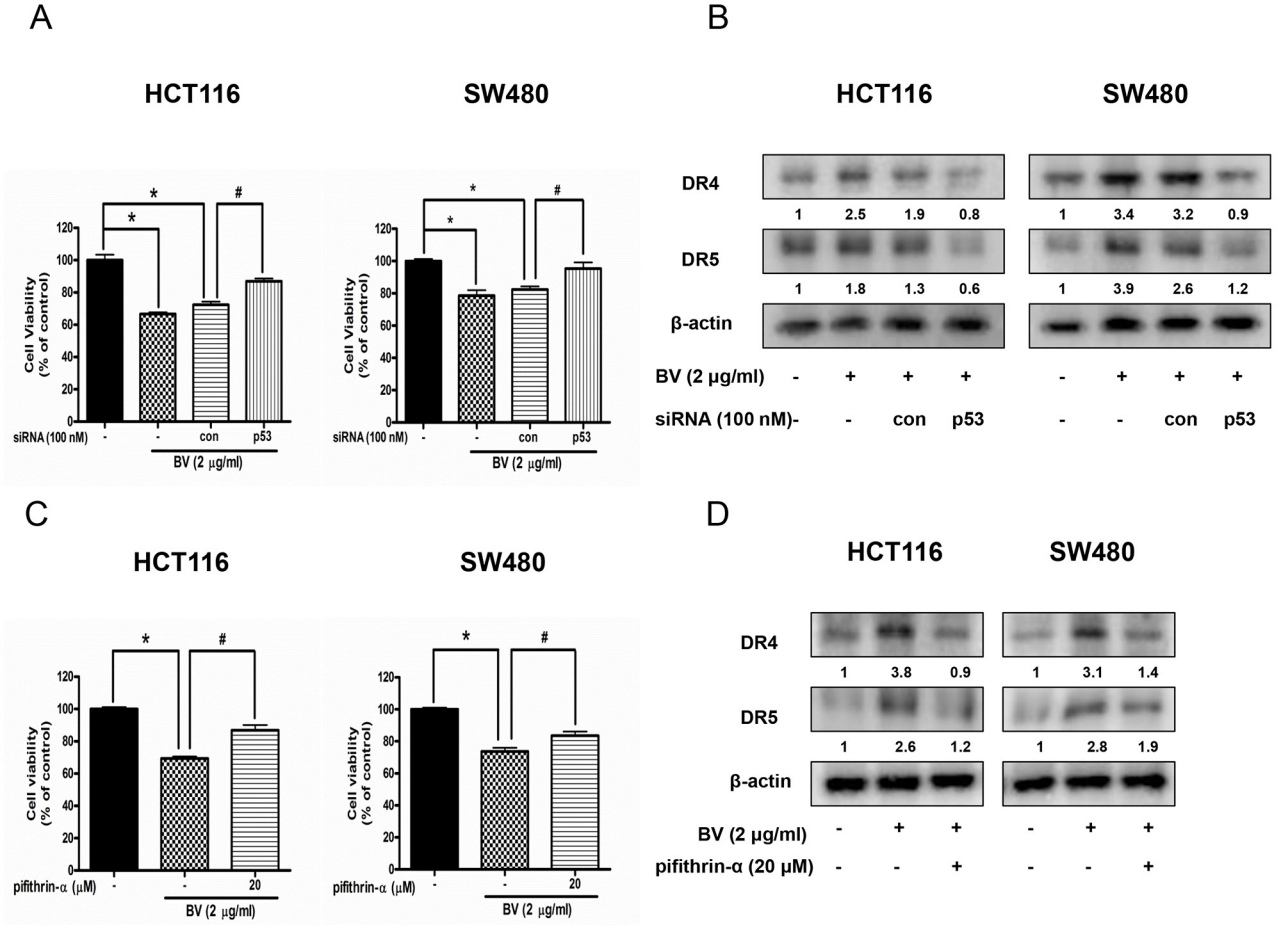
Effect of p53 siRNA transfection or p53 inhibitor (pifithrin-α) on BV-treated colon cancer cell growth and expression of DR4 and DR5 **A.** Colon cancer cells were treated with non-targeting control siRNA and p53 siRNA (100 nM) for 24 h, and then were treated with BV (5 μg/ml) at 37°C for another 24 h. Cell viability was determined by MTT assay. Data was expressed as the mean ± S.D. of three experiments. *p < 0.05 indicates statistically significant differences from control cells. #*p* < 0.05 indicates significantly different from BV treated cells. **B.** Effect of p53 siRNA transfection on the expression of DR4 and DR5. Whole cell extracts were analyzed by Western blotting with antibodies against DR4, DR5 and β-actin (internal control). Values under Western band indicate the density of band. Each band is representative for three experiments. **C.** Cells were pretreated with p53 inhibitor, pifithrin-α (20 μM) for 1 h and then were treated with BV for 24 h. Cell viability was determined by MTT assay. *p < 0.05 indicates significantly different from control cells. #*p* < 0.05 indicates significantly different from BV-treated cells. **D.** Effect of pifithrin-α on the expression of DR4 and DR5. Cells were pretreated with pifithrin-α (20 μM) for 1 h and then were treated with BV for 24 h, and whole cell extracts were analyzed by Western blotting using DR4, DR5 and β-actin (internal control). Values under Western band indicate the density of band. Each band is representative for three experiments.

To determine whether p53-dependent apoptosis pathway is involved in BV-induced cell death or not, the HCT116 and SW480 colon cancer cells were transfected with p53 siRNA by a transfection agent. The cells were transfected with 100 nM p53 siRNA for 24 h, and then treated with BV (2 μg/ml) for 24 h. Knock down of p53 partially abolished the cell growth inhibitory effect of BV in HCT116 and SW480 (Figure 7A). Expression of DR4 and DR5 by BV was suppressed by transfection with p53 siRNA both in HCT116 and SW480 cells (Figure 7B). To further investigate whether p53 plays an important role on BV-induced cell growth inhibition and up-regulation of DR4 and DR5, we pretreated HCT116 and SW480 colon cancer cells with 20 μM pifithrin-α, a p53 inhibitor for 1 h, and then these cells were treated with BV (2 μg/ml) for 24 h to assess cell viability and DR4 and DR5 expression. As a result, p53 inhibitor partially abolished BV-induced cell growth inhibition (Figure 7C) and suppressed the BV-induced up-regulation of DR4 and DR5 (Figure 7D), suggesting that p53-dependent apoptosis pathway may be involved in BV-induced cell growth inhibition and up-regulation of DR4 and DR5.

### Structure of melittin and molecular binding btween NF-κB p50 and melittin

**Figure 8:**
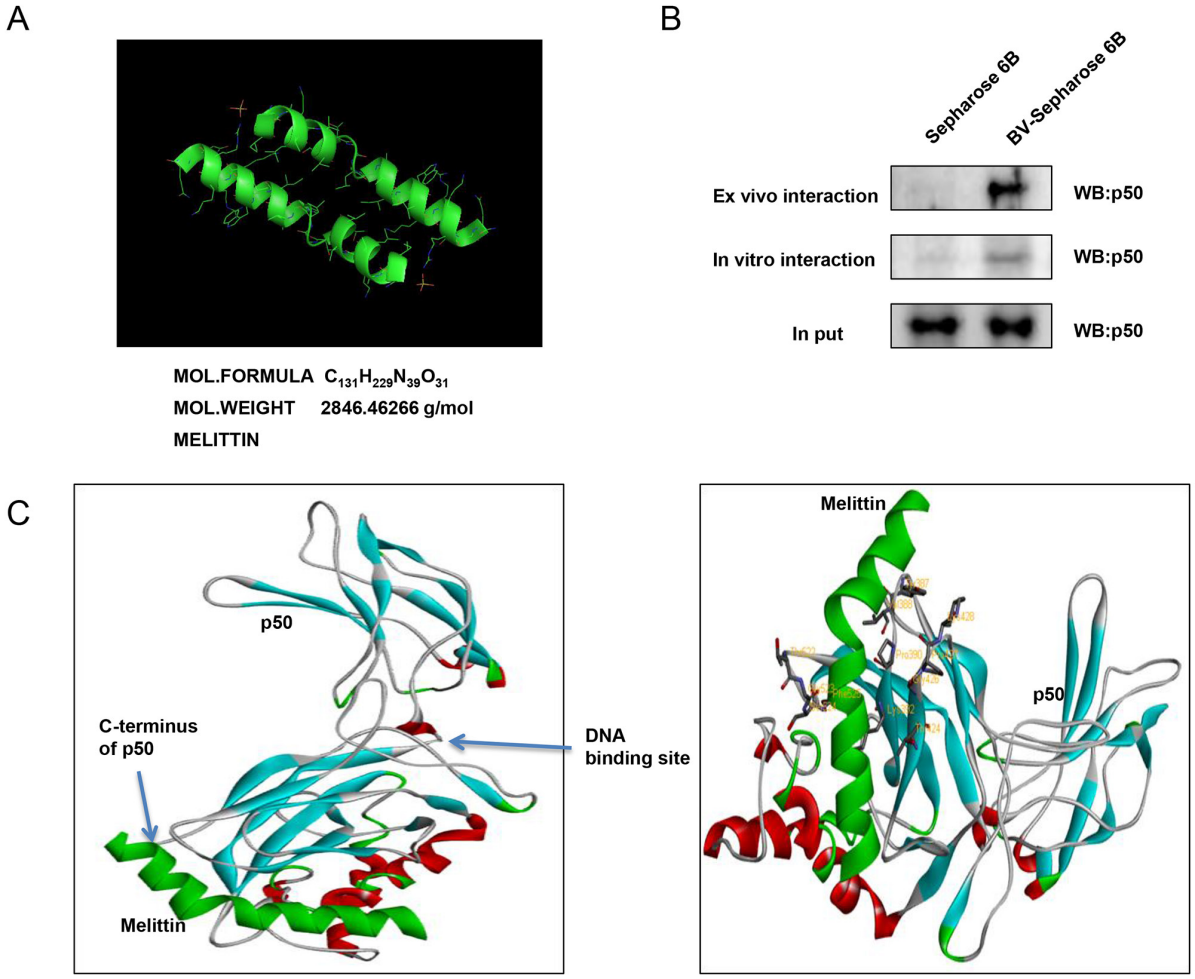
Structure of melittin, molecular binding between mellitin and NF-κB p50 **A.** Structure of mellitin. **B.** Pull down assay identifies the binding between mellitin and NF-κB p50. BV was conjugated with epoxy-activated Sepharose 6B. **C.** Docking model of mellitin with NF-κB p50 was determined as described in materials and methods.

The molecular binding between melittin, a major component of BV (Figure 8A) - p50 protein was assessed using pull-down assay. The interaction of melittin-Sepharose 6B beads with p50 was then detected by immunoblotting with p50 antibody. The results indicated that melittin bound with cell lysates containing p50 from SW480 cells and p50 recombinant protein (Figure 8B). To identify the binding site of melittin to p50, we performed computational docking experiments with melittin and NF-κB p50 subunit. Melittin bound somewhat near to the C-terminus of p50 where nuclear localization sequence (NLS) is located. Melittin bound to a pocket created by Tyr387, Val388, Pro390, Lys392, Thr424, Pro427, Lys428, Thr522, Ser524 and Phe525 of p50 (Figure 8C).

### Effect of BV on colon cancer tumor growth

**Figure 9:**
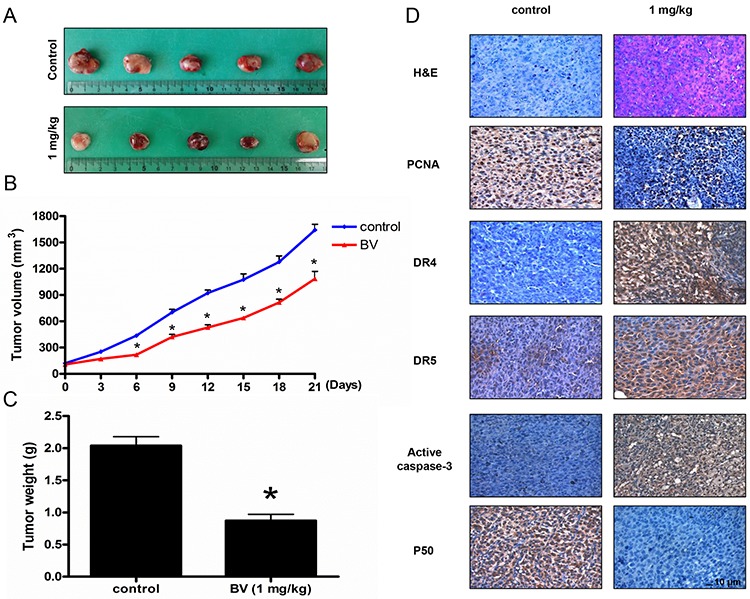
Effect of BV on the tumor growth, on the expression levels of proliferation, apoptosis regulatory proteins and NF-κB p50 subunit in immunohistochemistry Growth inhibition (as assessed by tumor volume and weights) of subcutaneously transplanted HCT116 xenografts mice treated with BV (1 mg/kg) twice a week for three weeks. Xenografted mice (*n* = 6) were administrated intraperitoneally with BV (1 mg/kg). **A.** Figure represents the tumor photographs at day 21. **B.** Tumor volumes were measured twice a week for three weeks. **C.** Tumor weights were measured at study termination on Day 21. Mean ± S.D. estimates of tumor volume and weight from 6 mice in each treatment. **D.** Immunohistochemistry was used to determine expression levels of H&E, PCNA, DR4, DR5, active caspase-3 and p50 in nude mice xenograft tissues by the different treatments as described in materials and methods. All values represent mean ± S.D. from 5 animal tumor sections. *p < 0.05 indicates significantly different from the control group. Bar indicates 10 μm.

To elucidate the anti-tumor effect of BV *in vivo*, the tumor growth on colon cancer cell xenograft bearing nude mice following BV treatments was investigated. In HCT116 xenograft studies, BV (1 mg/kg) was administrated intraperitoneally twice per week for 3 weeks to mice which have tumors ranging from 100 to 150 mm^3^. Tumor volume was measured twice a week, and all mice were sacrificed at the end of experiment when tumors were dissected and weighted. The inhibitory effect of BV on the growth of colon tumor was significant in xenograft model mice (Figure 9A). Tumor volume and weight were dose-dependently decreased (Figure 9B and 9C). The immunohistochemistry analysis of tumor section by hematoxylin and eosin staining and proliferation antibodies against PCNA staining revealed that BV (1 mg/kg) significantly suppressed tumor growth, and the expression level of DR4, DR5 and active caspase-3 was increased while the expression level of p50 was decreased in nude mice xenograft tissues (Figure 9D).

### Effect of BV on the expression of apoptosis regulatory proteins and DNA binding activity of NF-κB in colon tumor tissues

**Figure 10:**
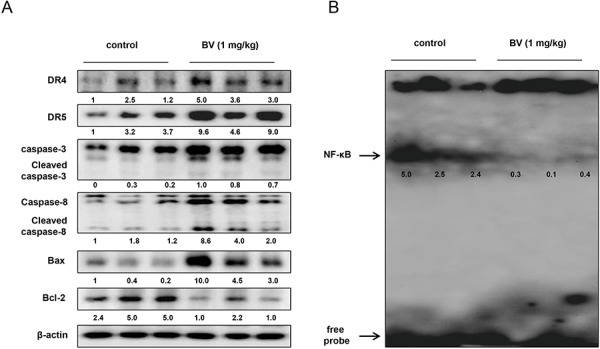
Effect of BV on the expression of apoptosis regulatory proteins and on the DNA binding activity of NF-κB *in vivo* **A.** Expression of apoptosis regulatory proteins was detected by Western blotting using DR4, DR5, Bax, Bcl-2, caspase-3, caspase-8 and β-actin (internal control) in xenograft tumor samples (3 samples/each group) as described in materials and methods. **B.** DNA binding activity of NF-κB was determined by EMSA in nucleus extract from xenograft tumor samples (3 samples/each group) as described in materials and methods.

To examine the relationship between colon tumor growth and apoptosis regulatory proteins as well as the DNA binding activity of NF-κB, we performed Western blotting and EMSA experiments. We found the expression of DR4, DR5, cleaved caspase-3, cleaved caspase-8 and Bax was increased, but the expression of Bcl-2 was decreased (Figure 10A). We also found the DNA binding activity of NF-κB was decreased in BV-treated colon tumor tissues (Figure 10B).

## DISCUSSION

Although it has been previously reported that BV can induce apoptosis in many cancer cell lines such as lung cancer, hepatocellular carcinoma, breast cancer, prostate cancer and ovarian cancer cell lines [29–33], there is no study on the effect of BV on colon cancer cell lines. In this study, we demonstrated that BV inhibited cancer cell growth in HCT116 and SW480 colon cancer cells through activation of DR4 and DR5 and suppression of NF-κB. Our present findings showed that BV inhibited the growth of HCT116 and SW480 colon cancer cells in a concentration dependent manner (0–5 μg/ml). However, the clear mechanism has not been found yet. The possible molecular targets of BV and its component for inhibition of cancer cell growth are numerous and various [34]. Our previous study showed that BV and its major component melittin inhibited cancer cell growth in prostate carcinoma cells through induction of apoptotic cell death *in vitro* as well as *in vivo* xenograft model via activation of caspase pathway through suppression of constituted activation of NF-κB activity [32], BV and melittin induced apoptotic cell death in ovarian cancer cells through enhancement of DR3, DR4 and DR6 expression and inhibition of STAT3 pathway [33], BV inhibited cancer cell growth in NSCLC cells through the induction of apoptosis via increase of DR3 expression and inhibition of NF-κB pathway [35]. Similarly, we found that BV inhibited the growth of colon cancer cells through activation of DR4 and DR5 and inhibition of NF-κB pathway. These data suggest that differential signals are involved in the inhibitory effect of BV on cancer cell growth depending on cancer cell types.

It was reported that cancer cell growth inhibitory effect was correlated with the down-regulation of various cell proliferative genes regulated by NF-κB [36]. In agreement with this notion, we found that BV suppressed DNA binding activity of NF-κB. Moreover, the decrease of NF-κB DNA binding activity was associated with the inhibitory effect of BV on the IκB phosphorylation and nuclear translocation of p50 and p65 in colon cancer cells. The present data showed that BV also suppressed the expression of anti-apoptotic proteins like Bcl-2, while it increased the expression of pro-apoptotic proteins such as Bax, caspase-3, caspase-8 and caspase-9 which are regulated by NF-κB. Thus, BV may induce an alteration of expression of apoptosis and anti-apoptosis regulatory proteins to provide a favorable circumstance for the cancer cells to reach death status by down-regulation of NF-κB. Enhanced cell growth inhibition was found to occur with the combination treatment with NF-κB inhibitor PAO (phenylarsine oxide, 0.1 μM) or p50 siRNA trasfection and BV. It may be because BV binds to p50, and prevents its translocation to nucleus. P50 siRNA may work in other step (may inhibit its expression). Two different mechanisms could work synergistically or additively. These data suggest that inhibition of NF-κB contributed to the BV-induced inhibitory effect of colon cancer cell growth. Therefore, it is possible that reduced NF-κB activity may be correlated with the inhibition of colon cancer cell growth by BV treatment.

Recent studies on the signaling mechanisms of the DRs have revealed that members of the NF-κB and caspase families are key regulators of cell death. Expression of DRs leads to activation of caspase-8, which induces activation of effecter caspase like caspase-3 that can active caspase-9 to cause apoptotic cell death [37, 38]. NF-κB is also involved in growth arrest and/or apoptosis by regulating the expression of various target genes such as Cyclin D1, Bax, caspase-3, caspase-9, Bcl-2, IAP and survivin [39, 40]. These results may suggest that the inhibition of NF-κB correlates with the increased expression of DRs. Numerous studies have demonstrated that natural compounds-induced apoptosis in cancer cells may be correlated to the increase of DR expression. Garicinol, a polyisoprenylated benzophenone derivative, derived from dried rind of the fruit Garcinia indica can potentiate TRAIL-induced apoptotic cell death of human colon cancer cell through up-regulation of DR4 and DR5 [16]. Quercetin also enhanced TRAIL-mediated apoptosis in colon cancer cells by inducing the accumulation of death receptors in lipid rafts [17]. Zerumbone enhanced TRAIL-induced apoptosis through the induction of death receptors in human colon cancer cells [18]. In our previous study, we demonstrated that the snake venom toxin from *Vipera lebetina turanica* induces apoptosis of colon cancer cells through reactive oxygen species (ROS) and c-Jun N-terminal kinases (JNK) dependent death receptor (DR4 and DR5) expression [15], and (E)-2,4-Bis(p-hydroxyphenyl)-2-butenal inhibits colon cancer cell growth via suppression of NF-κB and induction of DR6 [41]. Similarly, our results showed that the expression of DR4 and DR5 was increased in both HCT116 and SW480 colon cancer cells. We also observed that BV induced the increasing expression of DR downstream apoptotic proteins such as cleaved caspase-3, cleaved caspase-8, cleaved caspase-9 and Bax but decreased expression of Bcl-2 in colon cancer cells. Transcriptional regulation of DR4 and DR5 is complex, and multiple potential binding sites of various transcription factors, including p53, are present in the upstream region of DR4 and DR5 [42]. Our data showed that the increasing expression of p53 and p21 are both induced by BV. Thus, the induction of DR4 and DR5 by BV treatment may be dependent on p53 in HCT116 and SW480 colon cancer cells. Besides, co-treatment with BV and p53 siRNA or pifithrin-α, a p53 inhibitor partially abolished BV-induced cell growth inhibition and activation of DR4 and DR5. These data suggest that BV-induced apoptotic cell death and inhibitory effect of cancer cell growth might be correlated with the activation of p53 dependent DR4 and DR5 expression.

Docking model and pull-down assay showed that melittin, the major component of BV, directly bound with NF-κB p50 subunit to inhibit the DNA binding activity of NF-κB. Melittin bound to the nuclear localization sequence (NLS: Tyr387, Val388, Pro390, Lys392, Thr424, Pro427, Lys 428, Thr522, Ser524 and Phe525), which was near to the C-terminus of p50. The binding site of p50 to melittin was not as the same as our previous finding which showed that melittin bound to the DNA binding site of p50 [20]. However, it is noteworthy that blocking NLS is important for the translocation of p50 to nucleus inhibiting NF-κB activity [43]. Importin α3 is shown to directly bind to NLS of NF-κB p50 and p65 proteins, and IκBα inhibits nuclear translocation of NF-κB by inhibiting the binding of p65 homodimers and p50/p65 heterodimers to importin α3 [44]. The inducible nuclear translocation of NF-κB in Jukat T lymphocytes is significantly inhibited by a cell-permeable3 peptide which could bind to the NLS of the NF-κB p50 subunit [45]. Therefore, the present data indicated that BV effectively inhibited colon cancer cell growth through inactivation of NF-κB by directly binding with p50 NF-κB subunit. Taken together, our results provide the mechanistic evidence that BV treatment results in induction of apoptosis of colon cancer cells through up-regulation of DR4 and DR5 as well as suppression of NF-κB. Therefore, our results suggest that BV could be applicable as an anti-colon cancer agent.

## MATERIALS AND METHODS

### Materials

Dried BV was purchased from You-Miel Bee Venom Ltd. (Hwasoon, Jeonnam, Korea). The composition of the BV was as previously described [26]. Caspase-3, caspase-8, caspase-9 antibodies were purchased from Cell Signaling Technology Inc. (Beverly, MA). DR3, DR4, DR5, Fas, TRAIL, IκBα, phospho-IκBα, p50, p65, p21, p53, Bcl-2, Bax, Histone-H1 and β-actin antibodies were purchased from Santa Cruz Biotechnology, Inc. (Santa Cruz, CA). The cell culture materials were obtained from Invitrogen (Carlsbad, CA), and other chemical reagents were from Sigma Chemical Co.

### Cell culture

The HCT116 and SW480 colon cancer cell lines and FHC colon epithelial normal cells were obtained from American Type Culture Collection (ATCC). HCT116 was cultured in DMEM (Gibco, Life Technologies, Grand Island, NY) medium supplemented with 10% heat inactivated fetal bovine serum (FBS) and 100 units/ml penicillin, 100 μg/ml streptomycin (Invitrogen). SW480 was cultured in RPMI 1640 medium supplemented with 10% heat inactivated FBS,100 units/ml penicillin and 100 μg/ml streptomycin. FHC was cultured in DMEM: F12 (1:1) medium (ATCC) supplemented with 10% heat inactivated FBS, 25 mM HEPES, 100 units/ml penicillin and 100 μg/ml streptomycin. Cell cultures were then maintained in an incubator within a humidified atmosphere of 5% CO_2_ at 37°C.

### Cell viability assay

Colon cancer cells HCT116 and SW480 were plated in 96-well plates for 24 h, and then were treated with BV (0–10 μg/ml) for 24 h. After treatment, cell viability was measured by MTT [3-(4,5-Dimethylthiazol-2-yl)-2,5-Diphenyltetrazolium Bromide] assay (Sigma Aldrich, St. Louis, MO) according to the manufacturer's instructions. Briefly, MTT (5 mg/ml) was added and plates were incubated at 37°C for 4 h before dimethyl sulfoxide (100 μl) was added to each well. Finally, the absorbance of each well was read at a wavelength of 540 nm using a plate reader.

### Apoptosis evaluation

Colon cancer cells HCT116 and SW480 were cultured on 8-chamber slides. The cells were treated with BV (0–5 μg/ml) for 24 h, and then washed twice with phosphate buffered saline (PBS) and fixed by incubation in 4% paraformaldehyde in PBS for 1 h at room temperature. TdT-mediated dUTP nick and labeling TUNEL assays were performed by using the DeadEnd™ Fluorometric TUNEL System (Promega Corporation, Madison, USA) according to manufacturer's instructions. Total number of cells in a given area was determined by using DAPI staining. The apoptotic index was determined as the number of TUNEL-positive stained cells divided by the total cell number counted × 100%.

### Soft agar colony formation assay

Effect of BV on colony formation ability of the HCT116 and SW480 cells were assessed by soft agar colony formation assay. The assay was done in 6-well plates. In each well, 2 ml of 0.6% agar (in culture medium) was layered in the bottom followed by 3 ml of 0.3% agar as the top layer. Approximately 8 × 10^3^ cells were the plated over the top layer. The cells were treated with BV (0–5 μg/ml) and maintained at 37°C in a humidified 5% CO_2_ atmosphere for 10 days. Then the colonies were counted under a microscope.

### Western blot assay

Western blot assays were performed as previously described [26]. After a short washing in TBST, the membranes were immunoblotted with the following primary antibodies: caspase-3, caspase-8, caspase-9 (1:1000 dilutions; Cell Signaling, Beverly, MA) and p53, p21, DR3, DR4, DR5, Fas, TRAIL, Bax and Bcl-2 (1:1000 dilutions; Santa Cruz Biotechnology, Santa Cruz, CA). The blots were performed using specific antibodies followed by second antibodies and visualization by chemiluminescence (ECL) detection system.

### Electrophoretic mobility shift assay and supershift assay

The DNA binding activity of NF-κB was determined using an electrophoretic mobility shift assay (EMSA) performed as according to the manufacturer's recommendations (Promega). Supershift assay was performed on HCT116 cells, and a small volume of p50 antibody (1 μl) was added to the binding mix, and incubated at 37°C for 30 min before loading. The relative densities of the DNA–protein binding bands were scanned by densitometry using MyImage (SLB), and quantified by Labworks 4.0 software (UVP, Inc., Upland, CA).

### Transfection of siRNA

2 × 10^5^ cells were plated in 6-well plates and were transiently transfected with siRNA, using a mixture of siRNA and the WellFect-EX PLUS reagent in OPTI-MEM, according to the manufacturer's specification (WelGENE, Seoul, Korea). The transfected cells were treated with BV (2 or 5 μg/ml) for 24 h and then used for detecting cell viability and protein expression.

### Pull-down assay

Pull-down assay was performed as described elsewhere [27]. The proteins were then resolved by SDS-PAGE followed by immunoblotting with antibodies against p50 (1:1000 dilutions, Santa Cruz Biotechnology, Santa Cruz, CA).

### Docking experiment

Docking studies between NF-κB p50 and melittin were performed using Autodock VINA [28] as described elsewhere [27].

### Antitumor activity study in *in vivo* xenograft animal model

Eight-week-old male BALB/C nude mice were purchased from Orient-Bio (Gyunggi-do, Korea). The mice were maintained in accordance with the Korea Food and Drug Administration guidelines as well as the regulations for the care and use of laboratory animals of the animal ethics committee of Chungbuk National University (CBNU-278-11-01). The mice were held for 4 days after arriving before they were injected with cells. The last injection of BV was at the 18th day, and after 3 days the mice were euthanized at the 21th day. The mice were fed with mouse diet (S-11102 Altromin Moused Diet 1314 IRR) purchased from Samtako Inc. during the study. Human colon cancer cell line HCT116 cells were injected subcutaneous (1 × 10^7^ tumor cells/0.1 ml PBS/animals) with a 27-gauge needle into the right lower flanks in carrier mice. After 14 days, when the tumors had reached an average volume of 100–150 mm^3^, the tumor-bearing nude mice were intraperitoneally (i.p.) injected with BV (1 mg/kg dissolved in PBS) twice per week for 3 weeks. The group untreated was designed as the control. The weight and tumor volume of the animals were monitored twice per week. The tumor volumes were measured with vernier calipers and calculated by the following formula: (A × B^2^)/2, where A is the larger and B is the smaller of the two dimensions. At the end of the experiment, the animals were sacrificed. The tumors were separated from the surrounding muscles and dermis, excised and weighed.

### Immunohistochemistry

The animal tissues were fixed in 4% paraformaldehyde and cut into 5 μm sections using a freezing microtome (Thermo Scientific, Germany). The sections were stained with hematoxylin and eosin (H&E) for pathological examination. For immunohistological staining, tumor sections were incubated with primary antibody against PCNA, DR4, DR5, active caspase-3 and p50 (1:500, Abcam, Cambridge, UK). Immunohistochemistry experiment was performed as previously described [26].

### Statistical analysis

All statistical analysis was performed with GraphPad Prism 4 software (Version 4.03, GraphPad Software, Inc., San Diego, CA, USA). Group differences in the Figures 5, 6, 7 were analyzed using two-way analysis of variance (ANOVA) followed by Tuckey's test. Otherwise, data were analyzed by one-way ANOVA followed by Dunnett's test. All values are presented as mean ± S.D. Significance was set at *p* < 0.05 for all tests.

## Abbreviations

ATCC: American Type Culture Collection
Bax: Bcl-2-associated X protein
Bcl-2: B-cell leukemia/lymphoma-2
BV: bee venom
DAPI: 4
DMEM: Dulbecco's modified Eagle's medium
DR: death receptor
EMSA: electrophoretic mobility shift assay
FBS: fetal bovine serum
IAP: inhibitor of apoptosis protein
IHC: immunohistochemistry
JNK: c-Jun N-terminal kinases
MAPK: mitogen-activated protein kinase
NF-κB: nuclear factor κB
NLS: nuclear localization sequence
NSCLC: non-small cell lung cancer
PAO: phenylarsine oxide
PBS: phosphate buffered saline
PVDF: polyvinylidene fluoride
ROS: reactive oxygen species
SDS: sodium dodecyl sulfate
TRAIL: tumor necrosis factor-related apoptosis-inducing ligand
TUNEL: terminal deoxynucleotidyl transferase;
